# Evaluating the Magnolol Anticancer Potential in MKN-45 Gastric Cancer Cells

**DOI:** 10.3390/medicina59020286

**Published:** 2023-02-01

**Authors:** Mahsa Naghashpour, Dian Dayer, Hadi Karami, Mahshid Naghashpour, Mahin Taheri Moghadam, Seyed Mohammad Jafar Haeri, Katsuhiko Suzuki

**Affiliations:** 1 Department of Anatomical Sciences, Medical School, Arak University of Medical Sciences, Arak 38481-7-6341, Iran; 2 Cellular and Molecular Research Center, Medical Basic Sciences Research Institute, Ahvaz Jundishapur University of Medical Sciences, Ahvaz 61357-15794, Iran; 3 Department of Molecular Medicine and Biotechnology, Faculty of Medicine, Arak University of Medical Sciences, Arak 38481-7-6341, Iran; 4 Department of Basic Medical Sciences, Faculty of Medicine, Abadan University of Medical Sciences, Abadan 6313833177, Iran; 5 Department of Anatomical Science, Faculty of Medicine, Ahvaz Jundishapur University of Medical Sciences, Ahvaz 61357-15753, Iran; 6 Faculty of Sport Sciences, Waseda University, 2-579-15 Mikajima, Tokorozawa 359-1192, Japan

**Keywords:** gastric cancer, MKN-45, cisplatin, magnolol

## Abstract

*Background and Objectives:* Combination therapy improves the effect of chemotherapy on tumor cells. Magnolol, used in treating gastrointestinal disorders, has been shown to have anti-cancer properties. We investigated the synergistic effect of cisplatin and magnolol on the viability and maintenance of MKN-45 gastric cancer cells. *Materials and Methods:* The toxicity of magnolol and/or cisplatin was determined using the MTT technique. The trypan blue method was used to test magnolol and/or cisplatin’s effect on MKN-45 cell growth. Crystal violet staining was used to assess the treated cells’ tendency for colony formation. The expression of genes linked to apoptosis, cell cycle arrest, and cell migration was examined using the qPCR method. *Results:* According to MTT data, using magnolol and/or cisplatin significantly reduced cell viability. The ability of the treated cells to proliferate and form colonies was also reduced considerably. Magnolol and/or cisplatin treatment resulted in a considerable elevation in *Bax* expression. However, the level of *Bcl2* expression was dramatically reduced. *p21* and *p53* expression levels were significantly increased in the treated cells, while *MMP-9* expression was significantly reduced. *Conclusions:* These findings show that magnolol has a remarkable anti-tumor effect on MKN-45 cells. In combination with cisplatin, magnolol may be utilized to overcome cisplatin resistance in gastric cancer cells.

## 1. Introduction

Gastric cancer is the most common type of gastrointestinal cancer and the second leading cause of cancer death [1]. Each year, more than 1,000,000 new cases of gastric cancer are recorded [2]. Approximately 70% of these cases are noticed in developed countries, particularly in East Asia [3]. Surgery is the most common treatment for gastric cancer [4]. Chemotherapy is an efficient way to improve the effectiveness of surgery [5]. However, the case response to chemotherapy is predicted to be between 20% and 40%, with a survival time of 6 to 11 months after treatment [6].

Cisplatin is one of the most commonly used medications to treat gastric cancer [6,7]. However, cisplatin does not work for all patients [7]. Furthermore, high-dose chemotherapy has several adverse effects [8]. Chemotherapy resistance is one of the most challenging aspects of treating gastric cancer [9]. DNA/RNA damage repair, drug efflux, apoptosis suppression, and nuclear factor (NF)-κB activation are the four primary methods for preventing cell death after cisplatin treatment [10].

In previous investigations, chemical resistance has been linked to *Bcl-2* overexpression in patients with gastric cancer [11]. Combination therapy addresses critical pathways synergistically or additively and improves treatment efficacy [11]. In South Korea, China, and Japan, the Chinese herb *Magnolia officinalis* is commonly used as a local cure for gastrointestinal issues, coughs, anxiety, and allergic conditions [12]. Magnolol is a hydroxylated biphenyl chemical derived from the stem bark of *Magnolia officinalis* that is frequently used in East Asia to treat acute pain, cough, anxiety, and gastrointestinal issues [13]. Magnolol has been shown to have anti-inflammatory, antioxidant, and tumor-suppressive properties [14]. The inhibitory effect of magnolol on interleukin (IL)-1, tumor necrosis factor (TNF)-α, and IL-6 expression confirms its anti-inflammatory efficacy [15]. Magnolol was shown to have an apparent apoptotic impact in SGC-7901 human gastric cancer cells [16]. Magnolol and its methoxylated derivative, 2-O-methyl magnolol, were recently found to inhibit the proliferation, migration, and invasion of hepatocellular carcinoma cell lines by inducing p21 and p53 activation [17]. The anti-tumor effects of magnolol and 2-O-methyl magnolol in vivo were also established [18]. Magnolol inhibits the cell cycle in human gallbladder cancer cells at the G0/G1 phase [19].

According to several studies, magnolol’s metabolic effects are mediated through the NF-κB/MAPK, Nrf2/HO-1, and PI3K/Akt signaling pathways [20]. The two leading causes of cancer cells’ resistance to chemotherapy are invasion and metastasis [21]. Magnolol inhibits the invasive capability of MDA-MB cells via downregulating of the NF-κB/MMP-9 signaling pathway [22]. Magnolol’s anti-invasive and anti-metastasis properties may also be explained by its anti-angiogenesis activity, which is mediated through vascular endothelial growth factor (VEGF) suppression [23].

Given magnolol’s effectiveness in lowering cell growth, invasion, and metastasis, the question of whether magnolol can be utilized to reduce resistance to chemotherapeutic treatments arises [24]. In a recent study, magnolol was shown to be cytotoxic to oral squamous cells [25]. The findings also showed that magnolol therapy makes oral squamous cells more sensitive to cisplatin [25,26]. Chu and colleagues found that magnolol (80 µM) induced cytotoxic effects comparable to cisplatin at a dose of 25 µM in NSCLC cell lines [27]. The current research aims to evaluate the magnolol effects on cisplatin sensitivity in gastric cancer cells.

## 2. Materials and Methods

This experimental study was approved by the Ethics Committee of Arak University of Medical Sciences, Arak, Iran (Ethical code: IR.ARAKMU.REC.1400.032).

### 2.1. Reagent Preparation

Magnolol was purchased from Carbosynth Co. (Compton, Berkshire, UK). As a stock reagent, *Magnolol officinalis* alcoholic extract was dissolved in DMSO (dimethyl sulfoxide) at a 100 mM concentration and kept at −20 °C.

### 2.2. Cell Culture Protocol

The MKN-45 cell line (IBRC C10137) was purchased from the Iranian Biological Resource Center and cultured in RPMI medium containing 20% FBS and 1% penicillin/streptomycin. The cells were incubated at 37 °C with 5% CO_2_. The cells were passaged when confluency was reached at 80% [28] (Figure S1).

### 2.3. MTT Assay

The cells were cultured at 10^4^ cells/well density in a 96-well culture plate and incubated at 37 °C with 5% CO_2_ for 24 h. Afterward, the culture medium was replaced with 100 µL of 0.5 mg/mL MTT solution, and the plates were incubated for 4 h at 37 °C with 5% CO_2_ in the dark. Then each well received 100 µL of DMSO, and the plates were shaken for 15 min. The absorbance of the samples was measured at 570 nm using an ELISA reader (Bio-Rad, Berkeley, CA, USA). The viability of the cells was calculated using the formula: 100 − (absorbance test/absorbance control) × 100 [29]. The IC_50_ values were calculated using Prism software.

### 2.4. Study Design

The cells were divided into four groups. Group 1 (control group) consisted of MKN-45 cells that received no treatment. Group 2 consisted of MKN-45 cells that received magnolol. Group 3 received cisplatin, and group 4 was treated with a combination of magnolol and cisplatin. All groups were incubated at 37 °C with 5% CO_2_ for 24 h and subjected to cell proliferation assay, colony formation analysis, and real-time PCR.

### 2.5. Cell Proliferation Assay

Cells were plated in 6-well plates at a density of 10^5^ cells per well and incubated for 24 h at 37 °C with 5% CO_2_. The culture medium was replaced after 48 h of cisplatin and/or magnolol treatment. The cells were cultivated for 24, 48, 72, 96, and 120 h. After that, trypan blue staining was used to determine the number of viable cells. The formula [(number of viable cells in sample/number of viable cells in control) × 100] was used to determine cell proliferation.

### 2.6. Colony Formation Analysis

To undertake a colony formation analysis, the cells were trypsinized and grown at a density of 20,000 cells/well on a 12-well plate and incubated at 37 °C for 24 h with 5% CO_2_. Afterward, the cells were treated in triplicate with cisplatin and/or magnolol for 48 h. The cells were then cultured for seven days with a new culture medium. After incubation, the cells were washed twice with PBS, and a 1:7 mixture of methanol and cold acetic acid was added. The plates were incubated at room temperature for 20 min. The fixative solution was removed in the next step, and the cells were stained with 0.5% crystal violet. The cells were washed four times with distilled water and thoroughly dried at room temperature. The stained colonies were evaluated using an inverted microscope.

### 2.7. Real-Time PCR

The gene expression of the treated groups was evaluated using real-time PCR. The RNXTM reagent (Sinaclon, Tehran, Iran) was used to extract the RNA according to the manufacturer’s instructions. Based on the manufacturer’s suggestion, 1 µg of generated RNA was used for cDNA synthesis using a CycleScript cDNA synthesis kit (CycleScript RT PreMix Bioneer, Daejon, Republic of Korea). The real-time PCR reaction was performed using an Ampliqon RealQ Plus Master kit for SYBR Green I^®^ (Ampliqon, Copenhagen, Denmark) on a Lightcycler^®^ Detection System (Roche, New York, NY, USA). The genes and primers used in real-time PCR are listed in Table 1. The relative expression of the genes was compared using β-actin as the housekeeping gene. The reactions were prepared in a 20 µL mixture containing 10 µL Master Mix kit, 0.5 µL of each primer (200 nM), 3 µL cDNA (300 ng), and 7 µL nuclease-free water. The PCR protocol consisted of a 10 min denaturation at 95 °C followed by 45 cycles at 94 °C for 15 s and 60 °C for 30 s. Two separate reactions without cDNA or RNA served as negative calibrators. The gene expression of different groups was compared using the 2^−ΔΔCt^ technique. The MIQE (the minimum information for publication of quantitative real-time PCR experiments) guideline was followed for all qPCR studies.

### 2.8. Statistical Analysis

Statistical analysis was performed by GraphPad Prism 6.0 software. All analyses were done in triplicate. One-way ANOVA followed by Tukey post hoc analysis was used to assess the differences between various means. The difference between the two independent groups was determined using the *t*-test. All experimental data were presented as the mean ± SEM. The level of significance for all tests was set at *p* < 0.05.

## 3. Results

### 3.1. The Effects of Magnolol and/or Cisplatin on MKN-45 Cells’ Viability

**Figure 1:**
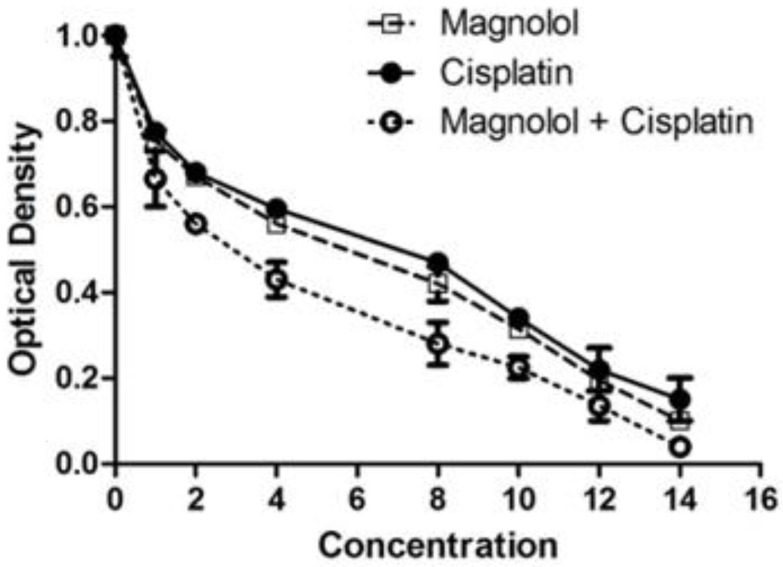
Changes in MKN Cells’ viability following magnolol and/or cisplatin therapy. The results of the MTT assay showed diminished optical density after treating of MKN-45 cell line with magnolol and/or cisplatin.

According to MTT data, magnolol treatment reduced cell viability dose-dependently (Figure 1). The algorithm of cisplatin’s effect on MKN-45 cells was similar to that of magnolol (Figure 1). However, the combination of magnolol and cisplatin resulted in a substantially more significant reduction in cell viability (Figure 1). IC_50_ values of magnolol, cisplatin, and their combination were 6.53, 7, and 3.25 µM, respectively.

### 3.2. The Effects of Magnolol and/or Cisplatin on MKN-45 Cells’ Proliferation

**Figure 2:**
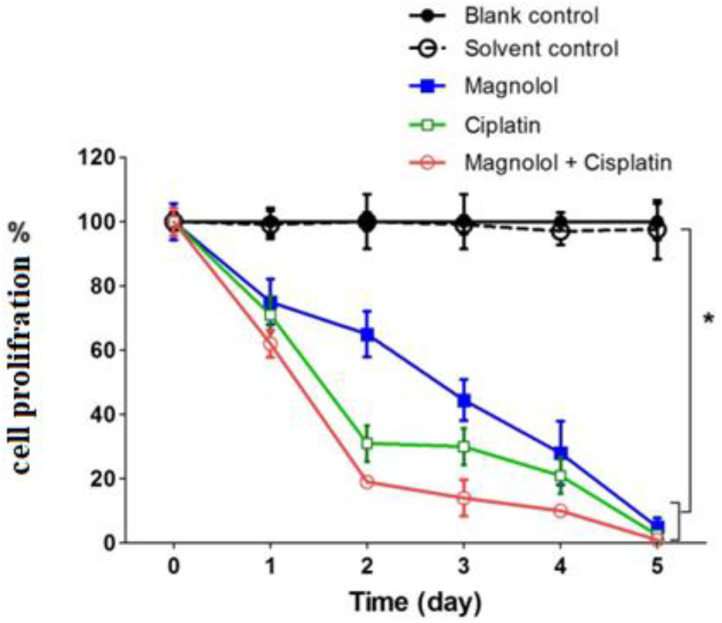
Proliferation assay following treatment of MKN-45 cells with magnolol and/or cisplatin. MKN-45 cells were treated with magnolol and/or cisplatin for 24 h. Then, the proliferation rate was calculated by counting the number of viable cells during five days compared to the control group (*: A significant difference between the cell proliferation % of the control group and other groups.)

According to the results of proliferation analysis, the cell growth rate was reduced to 62% and 59% of the control group after 24 h of treatment with magnolol and cisplatin, respectively (*p* < 0.05). However, the combination of the two medications decreased cellular proliferation by 55.5% compared to the control (*p* < 0.05). The tendency of lower cell proliferation in all treatment groups relative to the control group persisted until the fifth day of therapy. In the groups treated with magnolol, cisplatin, and magnolol plus cisplatin for five days, the proliferation amount reduced to 38.0%, 36.5%, and 36.7% of the control, respectively (*p* < 0.05) (Figure 2).

### 3.3. The Effects of Magnolol and/or Cisplatin on the Ability of MKN-45 Cells to Form Colonies

**Figure 3:**
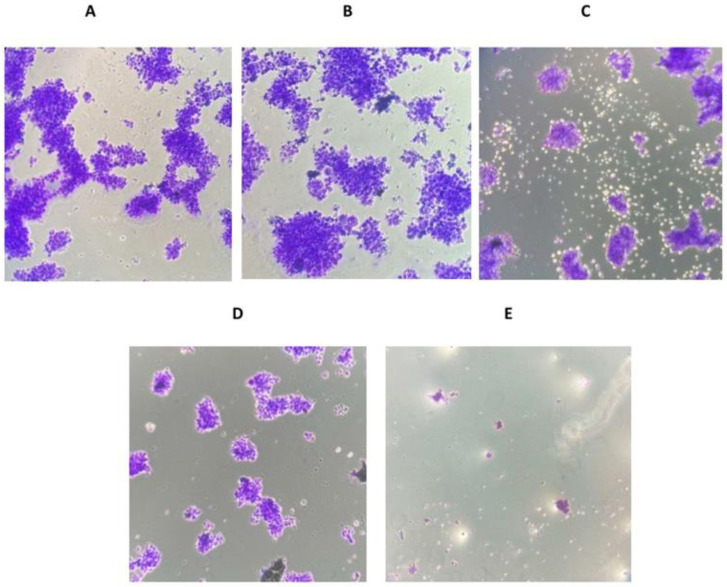
The effect of magnolol and/or cisplatin on colony formation in MKN-45 cells. (**A**) control group 1 (no treatment), (**B**) control group 2 (treated with PBS), (**C**) treated with magnolol, (**D**) treated with cisplatin, (**E**) treated with magnolol and cisplatin.

Following magnolol injection, the ability of MKN-45 cells to form colonies was significantly reduced. The cells that received cisplatin therapy showed a more significant decrease in colony formation. The cells that were given a combination of magnolol and cisplatin had the least ability to form colonies (Figure 3A–E).

### 3.4. The Effects of Magnolol and/or Cisplatin on the Expression of Apoptosis-Dependent Genes

**Figure 4:**
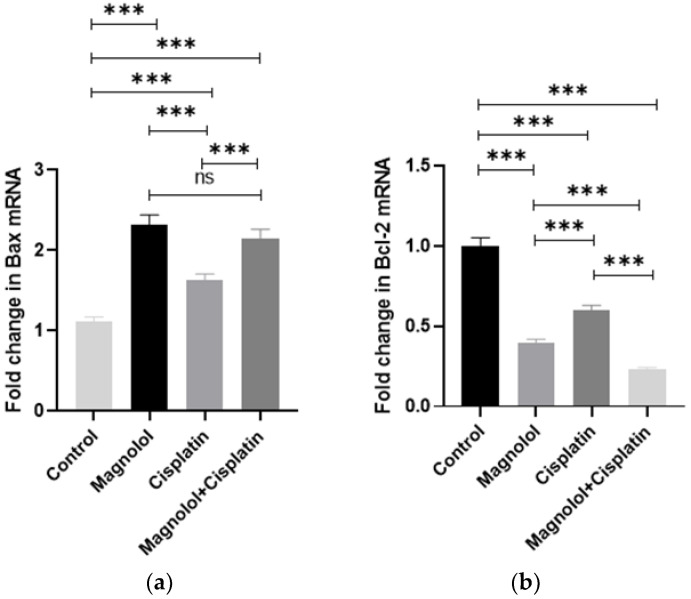
Changes in apoptotic-related genes following magnolol and/or cisplatin therapy. (**a**) A substantial increase in *Bax* expression after magnolol and/or cisplatin application. (**b**) A significantly decreased level of *Bcl2* expression after magnolol and/or cisplatin application. β-actin was used as the reference gene for normalizing the relative quantitative expression of target genes. All experiments were performed in triplicate. *** *p* < 0.001, ns: not significant.

Real-time PCR data revealed increased *Bax* expression following magnolol and/or cisplatin treatment. The group treated with magnolol showed the maximum increased level of *Bax* expression compared to the control group. In addition, a substantial increase in *Bax* expression in the groups treated with cisplatin or cisplatin + magnolol was noted (*p* ˂ 0.001). The result showed no significant increase in *Bax* expression in the magnolol-treated group compared to the magnolol + cisplatin-treated group (*p* > 0.05). However, a substantial increase in *Bax* expression was noted in the magnolol + cisplatin-treated group compared to the cisplatin-treated group (*p* ˂ 0.001) (Figure 4a). In all treated groups, *Bcl2* expression was significantly reduced compared to the control group (*p* ˂ 0.001). The group that received cisplatin with magnolol had the lowest level of *Bcl2* expression. The group that received magnolol + cisplatin showed a considerable reduction in *Bcl2* expression compared to those that received magnolol or cisplatin alone (*p* ˂ 0.001) (Figure 4b).

### 3.5. The Effects of Magnolol and/or Cisplatin on the Expression of Cell Cycle Regulator Genes

**Figure 5:**
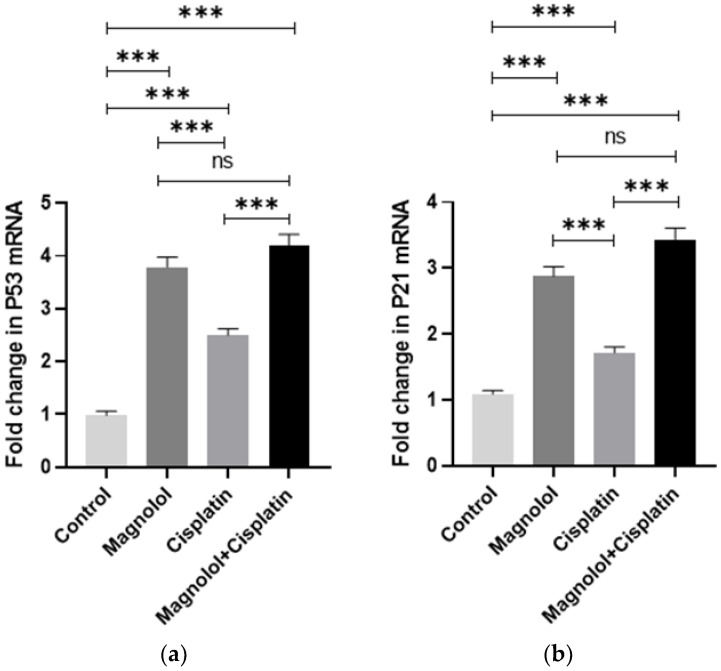
Changes in cell cycle regulator genes following magnolol and/or cisplatin therapy. (**a**) All treated groups showed significantly elevated levels of *p53* compared to the control group. The group that received a combination of magnolol and cisplatin had the maximum expression of *p53*. (**b**) All treated groups showed significantly elevated levels of *p21* compared to the control group. The group that received a combination of magnolol and cisplatin had the maximum expression of *p21*. β-actin was used as the reference gene for normalizing the relative quantitative expression of target genes. All experiments were performed in triplicate. ns: not significant. *** *p* < 0.001.

Magnolol and/or cisplatin treatment induced a significant increase in *p53* expression in comparison with the control group (*p* < 0.001) (Figure 5a). The group treated with magnolol + cisplatin showed the most significantly increased level of *P53*. There was no significant difference in P53 expression between the group that received magnolol alone and those treated with magnolol + cisplatin (*p* > 0.05). However, the combination therapy induced a significant elevation in *P53* expression compared with the group treated with cisplatin alone (*p* < 0.001) (Figure 5a). *p21* expression changes followed the same pattern as *p53* expression changes after magnolol and/or cisplatin treatment (Figure 5b).

### 3.6. The Effects of Magnolol and/or Cisplatin on the Expression of Extracellular Matrix Remodeling Gene Expression

**Figure 6:**
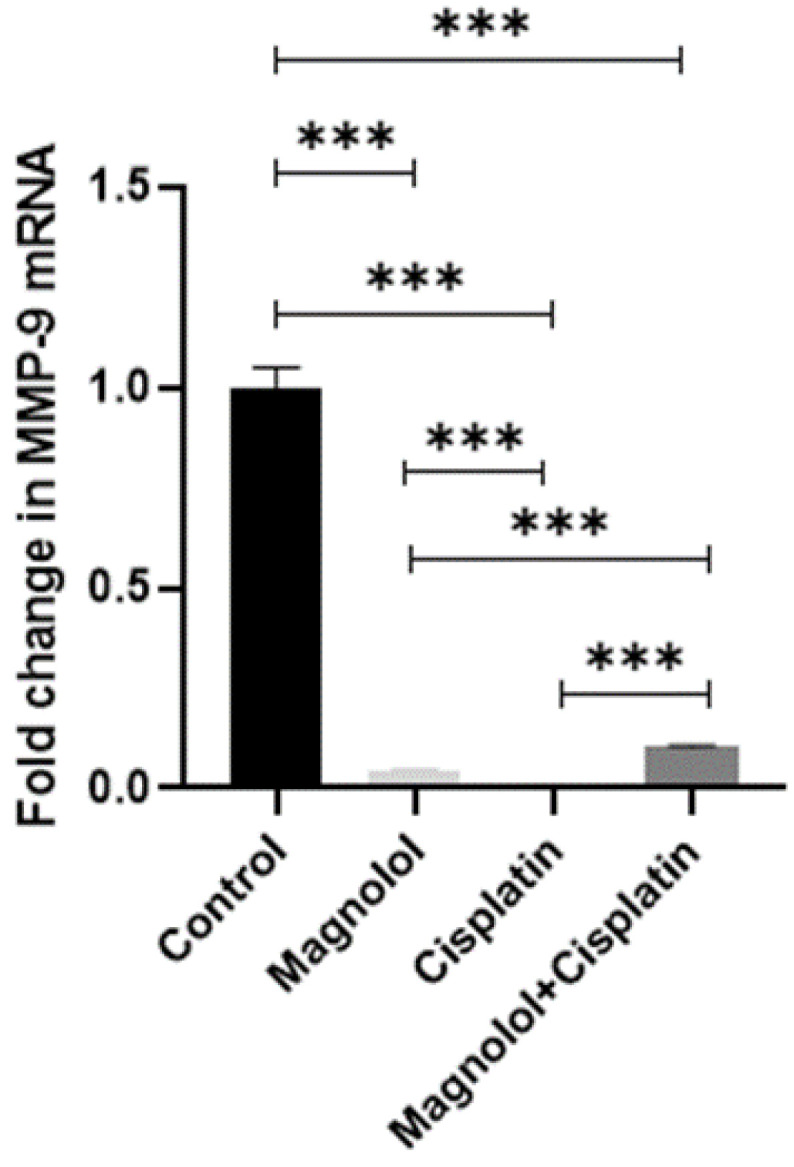
Changes in *MMP-9* wxpression following magnolol and/or cisplatin therapy treatment of MKN-45 cells with magnolol and/or cisplatin resulted in a substantial decrease in *MMP9* expression compared to the control group. β-actin was used as the reference gene for normalizing the relative quantitative expression of target genes. All experiments were performed in triplicate. *** *p* < 0.001.

*MMP-9* showed significantly reduced expression in the groups who received magnolol and/ or cisplatin (*p* < 0.001) (Figure 6). The group that received cisplatin presented the lowest expression of *MMP-9*. There was a significant difference between the treated groups in *MMP9* expression (*p* < 0.001) (Figure 6).

## 4. Discussion

This study aimed to see if magnolol could improve cisplatin cytotoxicity in gastric cancer cells. Cisplatin is commonly used to treat progressive gastric cancer [30]. However, cisplatin therapy has some unfavorable and harmful effects on normal cells [31]. Cisplatin induces unwanted apoptosis in blood, nerve, stomach, and kidney cells. Cisplatin binds to purine residues, causes DNA damage, and inhibits the cell cycle [27]. Cisplatin induces reactive oxygen species production and lipid peroxidation, resulting in unwanted ototoxicity. Another significant issue in gastric cancer treatment is cancer cells’ resistance to cisplatin [32]. Some evidence suggests that chemotherapy treatments without cisplatin considerably enhance survival time, progression-free survival, and response rate in gastric cancer patients [33]. In this regard, scientists worldwide are looking for natural compounds with anti-tumor properties. Some studies propose cisplatin and natural compound combination therapy to avoid the harmful effects of cisplatin [34]. Magnolol’s anti-cancer activity has been proven in several earlier investigations of various cancers [35]. Rasul et al. showed magnolol-induced apoptosis in SGC-7901 human gastric cancer cells.

This study investigated the effect of magnolol with or without cisplatin on MKN-45 gastric cancer cells in vitro. According to our findings, magnolol and cisplatin inhibited MKN-45 cell survival, proliferation, and colony formation ability in a dose-dependent way. Futhermore, the combination of magnolol and cisplatin had a substantially more significant death effect on cells [36]. According to Hyun et al. study, magnolia extract suppressed the survival of cervical cancer cells [37]. Regarding Ong et al.’s study results, magnolol inhibited the growth, colony formation, and proliferative capacity of non-small cell lung cancer cells [38]. Jian et al. found that treating human A549-bearing nude mice with honokiol, a bioactive component derived from magnolia, suppressed tumor growth considerably. When honokiol was combined with cisplatin, its anti-tumor efficacy was significantly increased [39]. In the next step, we investigated the mechanism by which magnolol lowers the viability of MKN-45 cells. In this regard, changes in apoptosis-related genes were studied.

The data demonstrated a noticeable increase in *Bax* expression and a significant decrease in *Bcl2* expression following magnolol and/or cisplatin treatment. The data from Park et al.’s study showed an increase in *Bax* expression and a reduction of *Bcl2* expression following the treatment of colon cancer cells with magnolol. According to their findings, magnolol causes cytochrome c movement from the mitochondria to the cytoplasm, leading to caspase-3 activation and cell death [40]. According to Rasul et al., magnolol has an apparent apoptotic effect mediated by an increase in *Bax*/*Bcl2* and caspase-3 expressions. The researchers reported that magnolol-induced apoptosis in gastric cancer cells could be related to increased mitochondrial membrane permeability and caspase pathway activation or decreased PI3K/Akt [41]. The exact mechanism by which magnolol reduces the viability of cancer cells is unclear [42]. Tsai et al.’s study revealed that magnolol inhibits apoptosis in non-small cell lung cancer cells via caspase-independent mechanisms. According to the observations, magnolol enhances the release of *Bid, Bax*, and cytochrome c from mitochondria. However, magnolol does not affect the expression of caspase-3, -8, or -9. The researchers concluded that magnolol inhibits non-small cell lung cancer cell proliferation by suppressing the PI3K/AKT and ERK1/2 pathways [43].

The next step of our study was to see how magnolol treatment affected the expression of cell cycle-regulating genes. In the groups treated with magnolol and/or cisplatin, we found a significant increase in *p53* and *p21* expression. The *p53* tumor suppressor gene is a critical transcription factor that controls angiogenesis, cell cycle, and DNA repair gene expression [44]. *p53* stops the cell cycle at stage G1 in response to DNA damage. *p53* is critical for genome preservation and is required for maintaining the shape and number of genomes [45]. Alterations in the *p53* gene were found in 77% of gastric cancer patients [46]. *p21* is defined as a downstream target of the tumor suppressor *p53*. *p53* binds to the *p21* promoter and stimulates its transcription [47]. *p21* is a cyclin-dependent kinase inhibitor that controls the activity of several cyclins and cyclin-dependent kinases involved in cell cycle regulation [48]. *p53* and *p21* have been suggested as critical biomarkers in cancer diagnosis. The findings of a clinical investigation showed that *p53* overexpression was associated with an improved response to chemotherapy in individuals with gastric cancer [49]. The increased levels of *p53* and *p21* expression identified in our study revealed that magnolol or magnolol plus cisplatin treatment could activate cell cycle arrest.

These results indicate that magnolol increases cellular sensitivity to cisplatin in MKN-45 cells. Hsu et al. discovered that magnolol treatment of colon cancer cells results in a significant increase in *p21* expression via the Ras/Raf-1-mediated activation of ERK [50]. According to a study by Shen et al., magnolol decreases the mitotic phase and the progress of G2/M in a dose-dependent manner [51]. According to the findings of a study on the effect of magnolol on rat xenograft cells, magnolol inhibits and dramatically suppresses β-catenin nuclear transfer and attaches the TGF β-catenin complexes to the DNA-bound axis in the nucleus [51]. In SW480 and HCT116 human colon cancer cells, these processes result in decreased regulation of target β-catenin/TCF genes such as c-myc, MMP-7, and plasminogen activator urokinase. In HCT116 nude mouse xenograft cells, magnolol also slows invasion and demonstrates anticancer efficacy [51]. In human umbilical vein endothelial cells, magnolol also suppresses the proliferation of basal fibroblast growth factor and the creation of capillary tubules [52]. The findings of the Zhou et al. investigation showed that magnolol increased cell cycle arrest at the G2/M phase by elevating *p21* and *p53* expression and reducing cyclin-B1 and CDK-1 expression [53].

The final phase of our research examined the effects of magnolol/cisplatin treatment on MKN-45 cell migration and invasion. The findings revealed that magnolol and cisplatin dramatically reduced gastric cancer cell migration, which correlated to a reduction in *MMP-9* expression. This finding is similar to earlier cellular studies that showed magnolol promotes cancer cell migration and invasion by reducing the NF-κB signaling pathway and MMP activity in breast cell lines and cholangiocarcinoma, respectively [54]. According to previous research, *MMP-9* is overexpressed in cancer cells [55]. It has been demonstrated that *p53* inactivation induces *MMP-9* production via enhancing glycolysis [56]. Hypoxia generally causes cell necrosis, leukocyte infiltration, and the release of TNF-α and IL-6 in tumor cells [57]. This condition causes *MMP-9* overexpression by activating the PI3k/AKT and MAPK pathways [58]. Furthermore, tumor hypoxia stimulates HIF-1 expression, increasing *MMP-9* activity [59]. The increase in *MMP-9* expression in tumor tissues plays a vital role in realizing the metastatic process’s sequential phases [60]. Our study’s considerable reduction in *MMP-9* suggested that magnolol and/or cisplatin treatment might have an anti-invasive and anti-metastatic effect. According to Liu et al.’s report, magnolol inhibited breast cancer cell invasion by down-regulating NF-κB and MMP-9 [22]. According to Nagas et al., magnolol substantially lowers malignancy in the human fibrosarcoma cell line HT-1080 by reducing *MMP-9* activity [61]. Finally, our findings, which are comparable with evidence from previous in vitro and in vivo investigations, confirm magnolol’s anti-tumor activities in MKN-45 gastric cancer cells. Our results also indicate that magnolol improves cisplatin’s anti-tumor efficacy.

## 5. Conclusions

Using magnolol as a single drug or combined with cisplatin may be a novel treatment for gastric cancer patients. However, more in vivo and clinical research is needed to determine the precise effects of magnolol on normal and malignant cells. More investigation is recommended to determine the exact molecular mechanisms of magnolol on normal and cancer cells.

## Figures and Tables

**Table 1:** Characteristics of primers used in qPCR test.

Gene Name	Primer Sequence
β-actin-hum-F	CAGCCTCAAGATCATCAGCAATG
β-actin-hum-R	CATGAGTCCTTCCACGATACCA
Bax-hum-F	AAGAAGCTGAGCGAGTGTCT
Bax-hum-R	TGCCGTCAGAAAACATGTCAG
MMP-9-hum-F	TAAGGAGTACTCGACCTGTACCA
MMP-9-hum-R	GAGGAACAAACTGTATCCTTGGTC
Bcl-2-hum-F	GGATGCCTTTGTGGAACTG
Bcl-2-hum-R	CAGCCAGGAGAAATCAAACAG
P53-hum-F	CAGACCTATGGAAACTACTTCCTG
P53-hum-R	ATTCTGGGAGCTTCATCTGGA
P21-hum-F	ATGTGGACCTGTCACTGTCTT
P21-hum-R	CGTTTGGAGTGGTAGAAATCTGTC

## Data Availability

The data presented in this study are available upon request from the corresponding author.

## Supplementary Materials

The following supporting information can be downloaded at https://www.mdpi.com/article/10.3390/medicina59020286/s1, Figure S1: The changes in morphological characteristics of MKN-45 cells during magnolol and/or cisplatin treatment. A. MKN-45 gastric cancer cells without treatment (control group). B. Magnolol therapy induced a significantly reduced number of viable cells. C. Cisplatin therapy resulted in a reduced number of viable cells and colonies. D. Cisplatin and magnolol combination therapy had the maximum inhibitory effect on MKN-45 cell viability. Figure S2: The results of electrophoresis. The real-time PCR products were loaded on 1% agarose gel and detected using a transilluminator (Jal Doc, Iran).
