# Supplementary material for: Evaluating the Magnolol Anticancer Potential in MKN-45 Gastric Cancer Cells
Source: Medicina (Kaunas). 2023 Feb 1;59(2):286. doi: 10.3390/medicina59020286 (PMC9963572; doi:10.3390/medicina59020286)
Supplement: Supplementary file 1 [file medicina-59-00286-s001.zip › medicina-2168145-supplementary.pdf]

Supplementary Materials: Evaluating the Magnolol Anticancer Potential in MKN-45 Gastric Cancer Cells

Mahsa Naghashpour, Dian Dayer, Hadi Karami, Mahshid Naghashpour, Mahin Taheri Moghadam, Seyed Mohammad Jafar Haeri* and Katsuhiko Suzuki*

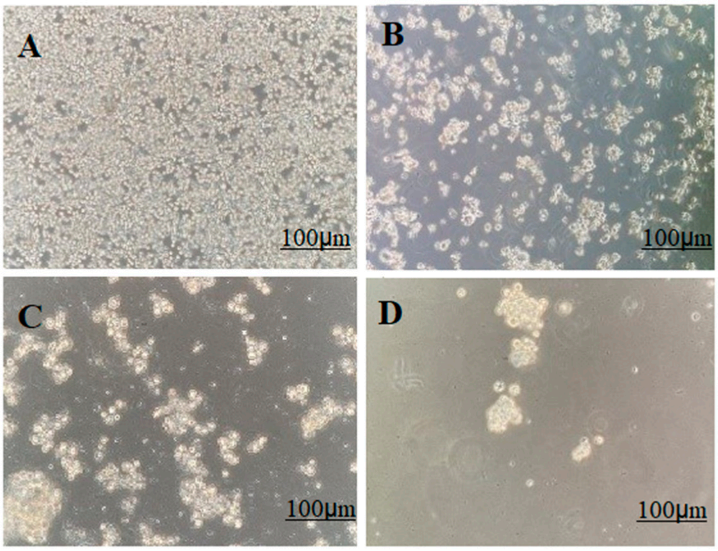

Figure S1: The changes in morphological characteristics of MKN-45 cells during magnolol and/or cisplatin treatment. A. MKN-45 gastric cancer cells without treatment (control group). B. Magnolol therapy induced a significantly reduced number of viable cells. C. Cisplatin therapy resulted in a reduced number of viable cells and colonies. D. Cisplatin and magnolol combination therapy had the maximum inhibitory effect on MKN-45 cell viability.

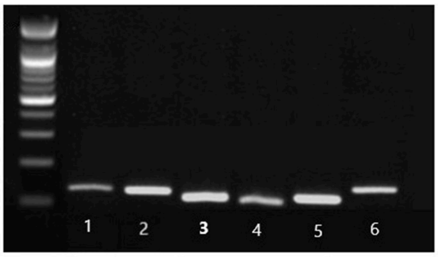

1	2	3	4	5	6
β-actin	BAX	P21	BCL2	MMP9	P53
145 bp	142 bp	136 bp	116 bp	127 bp	135 bp

Figure S2: The results of electrophoresis. The real-time PCR products were loaded on 1% agarose gel and detected using a transilluminator (Jal Doc, Iran).
